# Structural heart disease as the cause of syncope

**DOI:** 10.1590/1414-431X20176989

**Published:** 2018-03-01

**Authors:** R.B. Guimarães, V. Essebag, M. Furlanetto, J.P.G. Yanez, M.G. Farina, D. Garcia, E.D. Almeida, L. Stephan, G.G. Lima, T.L.L. Leiria

**Affiliations:** 1 Fundação Universitária de Cardiologia, Instituto de Cardiologia do Rio Grande do Sul, Porto Alegre, RS, Brasil; 2 Sacre Coeur Hospital of Montreal, University of Montreal, Montreal, Quebec, Canada; 3 McGill University Health Center Research Institute, McGill University, Montreal, Quebec, Canada

**Keywords:** Syncope, Hospitalization, Structural heart, Emergency

## Abstract

We described the clinical evolution of patients with structural heart disease presenting at the emergency room with syncope. Patients were stratified according to their syncope etiology and available scores for syncope prognostication. Cox proportional hazard models were used to investigate the relationship between etiology of the syncope and event-free survival. Of the 82,678 emergency visits during the study period, 160 (0.16%) patients were there due to syncope, having a previous diagnosis of structural heart disease. During the median follow-up of 33.8±13.8 months, mean age at the qualifying syncope event was 68.3 years and 40.6% of patients were male. Syncope was vasovagal in 32%, cardiogenic in 57%, orthostatic hypotension in 6%, and of unknown causes in 5% of patients. The primary composite endpoint death, readmission, and emergency visit in 30 days was 39.4% in vasovagal syncope and 60.6% cardiogenic syncope (P<0.001). Primary endpoint-free survival was lower for patients with cardiogenic syncope (HR=2.97, 95%CI=1.94-4.55; P<0.001). The scores were analyzed for diagnostic performance with area under the curve (AUC) and did not help differentiate patients with an increased risk of adverse events. The differential diagnosis of syncope causes in patients with structural heart disease is important, because vasovagal and postural hypotension have better survival and less probability of emergency room or hospital readmission. The available scores are not reliable tools for prognosis in this specific patient population.

## Introduction

Syncope is defined as transient loss of consciousness associated with an inability to maintain postural tone resulting from reduced blood flow in the reticular region in the brainstem. Syncope is an important public health problem, often disabling and may be the only warning before sudden cardiac death (1). Prevalence studies have indicated that 40% of the adult population has gone through a syncope episode, although the exact incidence is difficult to define, since many patients with syncope do not seek medical attention (2). The treatment of patients with syncope is a challenge for physicians, since the pathophysiology and causative factors are individualized. Although some of the causative factors have a good prognosis, about 11% are accompanied by severe outcomes such as acute myocardial infarction, cardiac arrhythmia, and sudden death (3–7). The diagnosis of a cardiac cause of syncope has important prognostic implications (1). Studies comparing mortality after syncope according to the probable mechanism have consistently shown that patients with a cardiac cause have a higher mortality than those with a noncardiac cause (8). In a study with more than 400 patients and a follow-up of more than 60 months, the mortality rate was 50% in patients with cardiac cause, compared to 31 and 24% for patients with a noncardiac or unknown cause, respectively (9).

Syncope in patients with structural heart disease is a serious finding. Although some patients may have relatively benign causes of syncope, in many cases the syncope may be related to arrhythmia and increased risk of subsequent life-threatening event (1–5).

Hospital admission for patients with heart diseases indicates a worse prognosis (6). Unplanned readmissions are associated with decreased quality of life, increased health-related costs, and, depending on the underlying heart disease, increased mortality (7).

Clinical risk scores have been developed to predict patients at high risk of sudden cardiac death presenting with syncope to emergency departments, such as the Boston Syncope Criteria, San Francisco Syncope Rule, Evaluation of Guidelines in Syncope Study (EGSYS), and Osservatorio Epidemiologico sulla Sincope nel Lazio (OESIL) scores. However, it is unclear if qualifying the syncope in patients with structural heart disease has the same prognostic information (3–5).

Our study sought to describe the clinical evolution of patients with structural heart disease presenting at the emergency department with syncope.

## Material and Methods

The study was approved by the Ethics and Research Committee of the Instituto de Cardiologia do Rio Grande do Sul (IC-FUC), Porto Alegre, RS, Brazil, and it is in compliance with the Declaration of Helsinki.

This cohort study included patients with structural heart disease diagnosed with syncope (Tenth Revision of the International Classification of Diseases - ICD-10 code R55) (8) in the emergency department of our institution from January 1, 2012, to December 31, 2013. Follow-up was done by telephone until July 1 2016. The study was conducted at the Emergency Department of the IC-FUC, Porto Alegre, RS, Brazil, a hospital specialized in heart diseases. The institution receives 50,000 emergency department visits annually.

### Inclusion and exclusion criteria

Patients with syncope presenting in the emergency department with a previous diagnosis of structural heart disease.

We excluded patients under 18 years old, pregnant women, cases with transient loss of consciousness misdiagnosed as syncope (such as those with the description of post-ictal state and seizures), individuals under the influence of illegal drugs or alcohol, patients diagnosed with acute myocardial infarction and stroke, and evident gastrointestinal bleeding during the initial evaluation or hypoglycemia. We also excluded patients who presented only with pre-syncope or dizziness.

### Definition of structural heart disease

Structural heart disease was defined as previous diagnosis of ischemic heart disease, heart failure, valve dysfunction (mild valve regurgitation was not included in this group), or primary myocardial structural disease (1).

### Definition of syncope

Syncope was defined according to the current guidelines as a non-traumatic transient loss of consciousness with loss of postural tone, fainting, and spontaneous recovery (8). Cardiogenic syncope was defined as a syncopal episode that had a clear cardiovascular cause such as: a) tachyarrhythmia or bradyarrhythmia identified during the conventional electrocardiography (EKG) recording, telemetry, pacemaker recordings or electrophysiological study; b) valvular heart disease (e.g., severe aortic stenosis, mitral stenosis, etc.) that required surgical or percutaneous intervention; c) ischemic heart disease not resulting in acute myocardial infarction; the patient required coronary angiography with or without percutaneous or surgical intervention; d) hypertrophic cardiomyopathy, arrhythmogenic right ventricle cardiomyopathy or other inherited myocardial disease when the episode was not typical for vasovagal syncope and that required further therapeutic action such as an implantable cardioversor/defibrillator; e) corrected congenital heart defects when the episode was not typical for vasovagal syncope.

### Data collection and chart review

To find all cases diagnosed with syncope during the study period, we used the electronic search engine of the emergency care system from our institution. The system is fully computerized and contains electronic medical records in which only one ICD-10 is allowed for the patient's chief complaint for the emergency department visit.

Data were entered in electronic medical records by the on-call attending cardiologist, including clinical history, physical examination, EKG results, laboratory tests, diagnosis, and clinical management. We adopted the recommendations of the recent consensus on the variables and endpoints for syncope study (1,7,8).

Demographic data (age and gender), clinical history (definition of syncope and its cause), data from syncope assessment, presence of structural heart disease, EKG (defining the cause of syncope, such as atrioventricular block, etc.), transthoracic EKG (left ventricular dysfunction), and information of hospitalization and reassessment at the emergency room were evaluated by a trained cardiologist and reviewed by an electrophysiologist.

A senior cardiologist performed the clinical assessment and chart review of all patients using our electronic medical record system. In addition to the initial medical evaluation, we investigated the occurrence of major adverse cardiac outcomes during follow-up.

Participants were contacted by telephone after stratification of the syncopal episode to investigate the occurrence of any additional adverse clinical outcomes outside the hospital.

### Outcomes

The primary outcomes were death, unscheduled emergency room visit, and hospital readmission for any cause during the follow-up.

### Risk stratification of syncope

During chart review, all included patients were classified into the different available risk scores for syncope (3–5). We must stress that these scores where not used in the clinical visit by the on-call physician and did not define the clinical conduct of each case. The OESIL, San Francisco, EGSYS, and Boston scores were calculated for each case. The OESIL (5) score ranges between 0 and 4, and is composed by the sum of the criteria: 1) age >65 years; 2) history of cardiovascular disease; 3) abnormal electrocardiogram; 4) syncope without prodrome. Mortality increase in one year was recorded according to the following: 0% score 0; 0.8% 1 point; 19.6% 2 points; 34.7% 3 points; 57.1% 4 points. Patients with moderate to high risk (score equal to 2 or higher) for presenting higher mortality in one year are eligible for hospitalization and investigation of etiological cause. The San Francisco rule (4) uses the following data: age >75 years, an abnormal ECG, hematocrit <30, a complaint of shortness of breath, and a history of heart failure. Any of these findings puts the patient at a higher risk for adverse events. The Boston syncope criteria utilizes eight categories of signs and symptoms that places patients with syncope at higher risk for adverse events or death in 30 days (3): 1) signs and symptoms of acute coronary syndrome; 2) signs of conduction heart disease; 3) worrisome cardiac history; 4) valvular heart disease by history or physical examination; 5) family history of sudden death; 6) persistent abnormal vital signs in the emergency department; 7) volume depletion, such as persistent dehydration, gastrointestinal bleeding, or hematocrit <30, and 8) primary central nervous system event. The EGSYS is a score that consists of the six (out of 52) items predictive of a cardiac cause for the syncope episode (9): palpitations preceding syncope (4 points), history of heart disease or abnormal electrocardiogram in the emergency department (3 points), syncope during effort (3 points) or while in supine position (2 points), precipitating or predisposing factors (1 point), and nausea or vomiting (1 point). A score of ≥3 is associated with a higher mortality than scores <3.

### Abnormal electrocardiogram

For the definition of abnormal EKG, the following were considered: left ventricle hypertrophy, presence of atrial fibrillation or flutter, supraventricular tachycardia, frequent supraventricular or ventricular extrasystoles (frequent was defined as triplets, bigeminy, trigeminy or more than 5 beats on a 12-s rhythm strip), sinus bradycardia or sinus pause (heart rate less than 60 bpm or a pause greater than 1.5 s), ventricular tachycardia, paced atrial or ventricular rhythm, atrioventricular or intraventricular conduction abnormalities (complete, first- or second-degree atrioventricular block, bundle branch block), severe axis deviation, electrically inactive area (abnormal Q waves) or repolarization abnormalities compatible with myocardial ischemia, a prolonged QTc interval, ventricular pre-excitation, and presence of the Brugada pattern. Early repolarization was considered normal, and only considered abnormal if the attending cardiologist described it as “suspicious malignant” early repolarization (e.g., ST-T downslope in inferior leads).

### Syncope prodromes

We searched the medical history for symptoms and signs that occurred before loss of consciousness. The prodromes were defined as nausea, diaphoresis, pallor, abdominal discomfort, yawning, blurred vision, tremors, palpitations, and lightheadedness moments before the loss of consciousness.

### Statistical analysis

The database was generated using IBM SPSS Statistics software, version 22.0.0 (IBM Corp., USA). Continuous variables are reported as means±SD or median with 95% confidence interval. Categorical variables are reported as absolute numbers and percentages. Univariate analysis was performed by the χ^2^ test and Fisher's exact test. The cumulative occurrence of major adverse cardiac outcomes was analyzed individually and with the Cox proportional hazard regression model, hazard ratios and confidence intervals (HR-95%CI) were reported. Additionally, we measured cumulative event-free survival of major adverse cardiac outcomes by the Kaplan-Meier method and compared unadjusted differences using the log-rank test. The significance level was set to P<0.05. To assess the predictive ability of the scores, the area under de ROC curve (AUC) was calculated.

## Results

**Figure 1.**
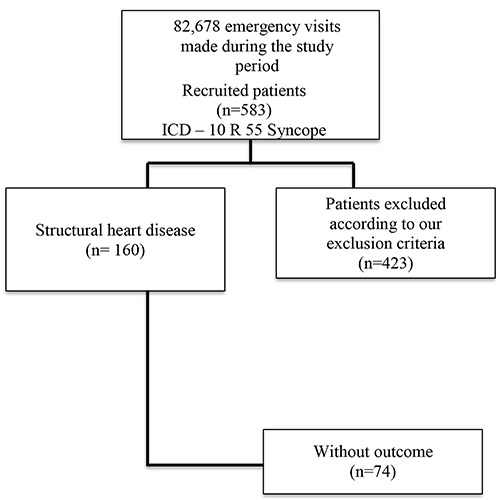
Study flow chart. ICD: International Classification of Diseases.

During the study period, there were 82,678 emergency visits including 583 individuals who received a diagnosis of syncope (0.7%). Four hundred and twenty-three patients were excluded based on the exclusion criteria (Figure 1).

One hundred and sixty patients remained in the cohort for having syncope and structural heart disease. The mean follow-up was 33.8±13.8 months. Mean age at the syncope event was 68.3 years and 40.6% of patients were male. Twenty-five percent of patients had a previous syncope episode. The diagnosis of syncope was vasovagal in 52, cardiogenic in 92, postural hypotension in 11, and due to unknown causes in 7 cases.

Prodromes occurred more frequently in those with vasovagal syncope (32 patients, 45%) and orthostatic hypotension (6 patients, 60%) when compared to those with cardiogenic syncope (40 patients, 43.5%; P<0.56). The causes of structural heart disease found in the four groups were ischemic heart disease (71.9%), valvular heart disease (14.4%), dilated cardiomyopathy (7.5%), hypertrophic cardiomyopathy (3.1%), combined ischemic and valvular heart disease (2.5%), and congenital heart disease (0.6%). The prevalence of causes did not differ among the different types of syncope (P=0.56; Table 1).

**Table 1.** Baseline characteristics for the two types of syncope.

	Vasovagal (n=52)	Cardiogenic (n=92)	P
Age (mean ± SD)	68.4±13	68.6±13	0.94
Female gender	26 (50)	33 (35.9)	0.098
Hypertension	47 (91.2)	82 (89.1)	0.81
Diabetes	11 (21.2)	20 (21.7)	0.59
Smoking	11 (21.2)	26 (28.3)	0.35
Previous stroke	4 (7.7)	7 (8.0)	0.83
Aortic stenosis	5 (8.8)	15 (17.2)	0.31
Heart failure	7 (13.5)	20 (21.7)	0.22
Abnormal EKG	29 (55.8)	70 (76.1)	0.015
EF <35%	2 (4.5)	16 (18.4)	0.095
Prodromes	32 (45)	40 (43.5)	0.56
Hospitalization	17 (32.7)	87 (94.6)	<0.001
Death	7 (13.5)	7 (7.6)	0.38

Data are reported as number and percent (chi-squared test). EKG: electrocardiogram; EF: ejection fraction.

**Figure 2.**
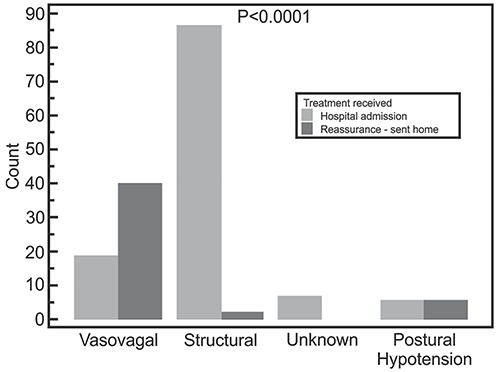
Causes of syncope and treatment received in the emergency room.

The on-call cardiologist made the decision for hospitalization in 98.9% of patients in the cardiogenic group, 100% in unknown causes patients, 31.6% in vasovagal syncope, and 50% for postural hypotension (P<0.001; Figure 2).

**Figure 3.**
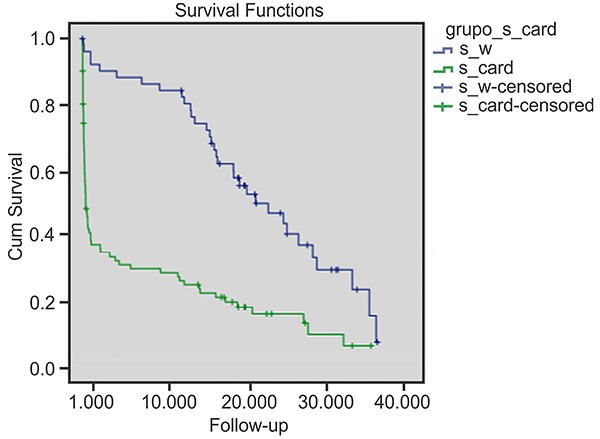
Primary outcome was a combination of death, unscheduled emergency room visit, and hospital readmission for any cause during follow-up. Cox cardiogenic syncope hazard ratio = 2.97 (1.94-4.55; P<0.001).

**Figure 4.**
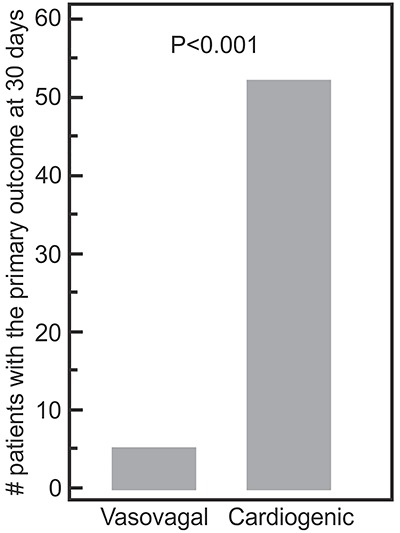
Primary outcome within 30 days in patients with vasovagal and cardiogenic syncope. The outcome was found in 39.4% of vasovagal and 60.6% of cardiogenic (P<0.001, chi-squared test).

Primary endpoint-free survival was lower in those with cardiogenic syncope compared to vasovagal syncope at all follow-ups (Figure 3). Deaths occurred in 4 (7%) patients with vasovagal syncope: 1 patient died of pneumonia, 1 from cancer complications, 1 from acute myocardial infarction, 1 from hemorrhagic stroke. Six (6.9%) died from cardiogenic syncope: 1 patient died of sepsis, 2 from complications of acute myocardial infarction, 1 from cardiac arrest in ventricular fibrillation, 1 patient died of external causes, 1 of unknown causes and no autopsy was performed. One patient (16.7%) with unknown syncope died of sepsis (P=0.65). Primary endpoint-free survival was lower for patients with cardiogenic syncope (HR 2.97, 95%CI=1.94-4.55, P<0.001) (Figure 4).

The OESIL, Boston, and San Francisco scores did not present predictive ability for adverse events during follow-up (Table 2).

**Table 2.** Predictive ability of the scores.

Score	AUC	95%CI	P
OESIL ≥2	0.53	0.43–0.65	0.532
San Francisco Syncope Rule ≥1	0.56	0.45–0.66	0.310
Boston Syncope Criteria ≥2	0.56	0.45–0.67	0.267
EGSYS ≥5	0.58	0.47–0.68	0.157

AUC: area under the ROC curve with 95% confidence intervals (CI). OESIL: Osservatorio Epidemiologico sulla Sincope nel Lazio; EGSYS: Evaluation of Guidelines in Syncope Study. The chi-squared test was used for statistical analysis.

## Discussion

Syncope is an important problem that cardiologists see on a daily basis in their clinical practices. Cases with cardiac causes of syncope are the most serious and are associated with the worst outcome (9–11). However, it is often unclear whether all patients with structural heart disease and non-cardiogenic syncope need to be admitted and whether their syncope indicates a worse long term prognosis (10,11).

Different from other studies in general hospitals that found 70% of syncope to be vasovagal and 15% cardiogenic, our cohort found 54% of syncope patients with a cardiogenic cause and only 35% considered vasovagal. This can be explained by the fact that our study was designed to include only patients with structural heart disease (12,13). Moreover, in our study, the median age ranged from 69–75 years between the groups and, as cited by Getchell et al. (14), previous data suggest that hospitalized patients and the elderly have a higher frequency of cardiogenic syncope.

Among the causes of structural heart disease, ischemic cardiomyopathy was the most prevalent (74%), followed by valve disease (17%) and dilated cardiomyopathy (7.5%). We found no significant difference in the causes of syncope according to type of heart disease.

There was no significant difference between groups regarding left ventricular ejection fraction and presence of abnormal EKG, and these variables did not predict the occurrence of the study endpoints (possibly because of insufficient power). However, other studies have found left ventricular ejection fraction and abnormalities on the EKG to be poor prognostic factors (1,15).

We found that prodromes were more prevalent but did not significantly predict the occurrence of events in patients with vasovagal syncope and orthostatic hypotension when compared to cardiogenic syncope and syncope due to an unknown cause. This is in accordance with the literature (1). The absence of prodromes is a reliable clue for the diagnosis of cardiogenic syncope as demonstrated by Sheldon et al. (11) and Alboni et al. (16).

Patients classification by the different scores for syncope prognosis did not show diagnostic performance and did not help differentiate patients with an increased risk of adverse events (primary endpoints) during follow-up. These scores have traditionally been validated for use in general emergency (3–5), a setting that includes patients with less comorbidities.

When analyzing the decision made during the initial clinical evaluation for admitting or dismissing the patient from the emergency department, we observed that 98.9% of patients in the cardiogenic group and 100% of patients with unknown cause underwent hospitalization, compared with only 31.6% of vasovagal syncope and 50% of postural syncope (Figure 2).

The diagnosis of the type of syncope was made by the attending physician based on clinical criteria. According to Thiruganasambandamoorthy et al. (17), the emergency department diagnosis between vasovagal and cardiogenic syncope, although subjective, had good inter-observer agreement and even in the absence of complementary diagnostic tests the diagnosis is made correctly most of the time.

Arnar (1), in a review, applied the Canadian Cardiovascular Society, American College of Cardiology, and European Society of Cardiology guidelines in patients that visited the emergency department to determine the effect on hospital admission rates. The data suggests that the application of syncope guidelines are unlikely to reduce admission rates, and that a lack of agreement exits among the different guidelines resulting in significant variation between warranted admissions (8–18).

In the assessment of primary outcomes-free survival, patients with cardiogenic syncope and syncope from unknown origin had significantly higher rates of adverse outcomes compared with vasovagal syncope and postural hypotension. Patients with postural hypotension and unknown cause were excluded from the analysis of predictors due to a reduced number of cases. The primary composite endpoint including death, readmission, and emergency visit in 30 days was 39.4% in vasovagal and 60.6% in cardiogenic (P<0.001; Figure 2). The medium follow-up was 33.8 months and during this period, 4 patients underwent myocardial revascularization surgery, 2 required electrical cardioversion, 5 underwent valve replacement, 12 required percutaneous revascularization, 19 required a retractive adjustment, 7 underwent electrophysiological study with induction of arrhythmia, 8 patients questioned cardio-defibrillator in emergency presenting 3 appropriate shocks. These data were not significantly different between the groups.

Few studies evaluated the evolution of vasovagal syncope in patients with structural heart disease. Sheldon et al. (19) reported that one of the most worrisome causes of syncope in patients with structural heart disease is ventricular tachycardia. Patients with a ventricular tachycardia cause have a significantly worse outcome than patients with vasovagal syncope, with 5-year outcome free survival of 54 and 84% for the ventricular tachycardia and vasovagal syncope groups, respectively (10).

We did not find a significant difference in mortality between groups. Nevertheless, our cohort experienced a high mortality rate (7%), which is not expected in patients with vasovagal syncope and structural heart disease (19). Hospital admission in patients with heart disease increases the risk of death, deteriorates the quality of life, and is a marker of worse prognosis. Our study showed that this might also be true when the hospitalization is due to syncope, even if from a vasovagal origin, considering our high death rate. Swindle et al. (20) reported that hospitalization is associated with increased risk of mortality and re-admission among patients hospitalized with heart failure similar to the risk observed in our patients.

### Limitations

Our study used a population recruited at a single cardiology reference hospital, therefore risk of selection bias cannot be ruled out as a function of a worse cardiovascular profile of our population compared to non-hospitalized people. We also had a lower prevalence of syncope in the emergency department than previously described in the literature. This may indicate that the ICD-10 for the final diagnosis of the syncope event was changed for some patients (e.g., ventricular tachycardia), precluding them from entering the study based on the ICD coding search.

Syncope in patients with structural heart disease predicts a worse prognosis in short and long term follow-up. The differential diagnosis between vasovagal, cardiogenic, and postural hypotension syncope is very important, because even in those patients with structural heart disease, vasovagal and postural hypotension have a better prognosis in terms of hospital readmission and death as a combined endpoint compared to cardiogenic syncope. The available scores are not useful in the presence of structural heart disease. Strategies to better identify the cause of the syncope are important in an already overloaded healthcare scenario of the emergency department because of prognostic and management implications even in patients with known structural heart disease.
